# Supplementary material for: Comparative genomics provides new insights into the diversity, physiology, and sexuality of the only industrially exploited tremellomycete: Phaffia rhodozyma
Source: BMC Genomics. 2016 Nov 9;17:901. doi: 10.1186/s12864-016-3244-7 (PMC5103461; doi:10.1186/s12864-016-3244-7)
Supplement: Additional file 6: — List of orphan genes with links to PFAM (related to Additional file 1: Table S1). (ZIP 1428 kb) [file 12864_2016_3244_MOESM6_ESM.zip › PFAM_UNIQUE/G01012_P.html]

#                                                                            --- full sequence --- -------------- this domain -------------   hmm coord   ali coord   env coord
  
# target name        accession   tlen query name           accession   qlen   E-value  score  bias   #  of  c-Evalue  i-Evalue  score  bias  from    to  from    to  from    to  acc description of target
  
#------------------- ---------- ----- -------------------- ---------- ----- --------- ------ ----- --- --- --------- --------- ------ ----- ----- ----- ----- ----- ----- ----- ---- ---------------------
  
G01012\_P             -           1268 DUF3348              PF11828.3    245      0.03   12.4   0.0   1   1   4.4e-05     0.087   10.9   0.0    98   148   589   639   566   644 0.83 - Protein of unknown function (DUF3348)  
G01012\_P             -           1268 PPR                  PF01535.15    31   2.1e-07   29.2   4.9   1   6      0.12        51    2.9   0.0     7    25   404   422   403   423 0.92 - PPR repeat  
G01012\_P             -           1268 PPR                  PF01535.15    31   2.1e-07   29.2   4.9   2   6     0.045        19    4.2   0.0     2    27   488   513   487   516 0.93 - PPR repeat  
G01012\_P             -           1268 PPR                  PF01535.15    31   2.1e-07   29.2   4.9   3   6       1.3   5.5e+02   -0.4   0.0     3    24   565   586   565   590 0.90 - PPR repeat  
G01012\_P             -           1268 PPR                  PF01535.15    31   2.1e-07   29.2   4.9   4   6   0.00028      0.12   11.1   0.2     5    28   649   672   648   673 0.94 - PPR repeat  
G01012\_P             -           1268 PPR                  PF01535.15    31   2.1e-07   29.2   4.9   5   6         2   8.5e+02   -1.0   0.0     3    29   812   838   811   839 0.87 - PPR repeat  
G01012\_P             -           1268 PPR                  PF01535.15    31   2.1e-07   29.2   4.9   6   6     0.014       5.8    5.8   0.0     2    25  1011  1034  1010  1038 0.88 - PPR repeat  
G01012\_P             -           1268 PPR\_1                PF12854.2     34   0.00033   18.8   4.6   1   4    0.0093        11    4.3   0.0    17    32   407   422   404   422 0.89 - PPR repeat  
G01012\_P             -           1268 PPR\_1                PF12854.2     34   0.00033   18.8   4.6   2   4   0.00025       0.3    9.3   0.3    13    34   650   671   649   671 0.90 - PPR repeat  
G01012\_P             -           1268 PPR\_1                PF12854.2     34   0.00033   18.8   4.6   3   4     0.062        74    1.6   0.0     2    14   675   687   674   688 0.94 - PPR repeat  
G01012\_P             -           1268 PPR\_1                PF12854.2     34   0.00033   18.8   4.6   4   4     0.063        76    1.6   0.1    20    31  1022  1033  1018  1034 0.88 - PPR repeat  
G01012\_P             -           1268 PPR\_2                PF13041.1     50   3.1e-08   32.1   0.9   1   5      0.17        99    1.7   0.0    10    28   404   422   403   423 0.91 - PPR repeat family  
G01012\_P             -           1268 PPR\_2                PF13041.1     50   3.1e-08   32.1   0.9   2   5      0.15        91    1.8   0.0     5    34   488   517   484   524 0.81 - PPR repeat family  
G01012\_P             -           1268 PPR\_2                PF13041.1     50   3.1e-08   32.1   0.9   3   5        10     6e+03   -4.4   0.0    23    34   547   558   544   559 0.83 - PPR repeat family  
G01012\_P             -           1268 PPR\_2                PF13041.1     50   3.1e-08   32.1   0.9   4   5   1.3e-07   7.9e-05   21.2   0.0     9    45   650   687   649   691 0.92 - PPR repeat family  
G01012\_P             -           1268 PPR\_2                PF13041.1     50   3.1e-08   32.1   0.9   5   5      0.54   3.2e+02    0.1   0.0    12    28  1018  1034  1010  1036 0.90 - PPR repeat family  
G01012\_P             -           1268 PPR\_3                PF13812.1     34     4e-06   25.4  11.1   1   7      0.02        12    5.2   0.2     2    31   487   516   486   519 0.86 - Pentatricopeptide repeat domain  
G01012\_P             -           1268 PPR\_3                PF13812.1     34     4e-06   25.4  11.1   2   7       3.3     2e+03   -1.7   0.0     4    22   565   586   565   590 0.66 - Pentatricopeptide repeat domain  
G01012\_P             -           1268 PPR\_3                PF13812.1     34     4e-06   25.4  11.1   3   7      0.37   2.2e+02    1.2   0.1     2    27   608   634   606   640 0.82 - Pentatricopeptide repeat domain  
G01012\_P             -           1268 PPR\_3                PF13812.1     34     4e-06   25.4  11.1   4   7   2.6e-05     0.015   14.2   0.1     6    34   649   678   649   678 0.93 - Pentatricopeptide repeat domain  
G01012\_P             -           1268 PPR\_3                PF13812.1     34     4e-06   25.4  11.1   5   7       7.8   4.7e+03   -2.9   0.0    19    32   735   748   729   749 0.79 - Pentatricopeptide repeat domain  
G01012\_P             -           1268 PPR\_3                PF13812.1     34     4e-06   25.4  11.1   6   7       1.1   6.8e+02   -0.3   0.0     4    28   812   836   809   839 0.88 - Pentatricopeptide repeat domain  
G01012\_P             -           1268 PPR\_3                PF13812.1     34     4e-06   25.4  11.1   7   7     0.021        13    5.1   0.0     3    26  1011  1034  1009  1040 0.90 - Pentatricopeptide repeat domain  
G01012\_P             -           1268 TPR\_14               PF13428.1     44   0.00048   19.3   3.6   1   4     0.048       5.4    6.7   0.0     2    31   487   516   486   530 0.83 - Tetratricopeptide repeat  
G01012\_P             -           1268 TPR\_14               PF13428.1     44   0.00048   19.3   3.6   2   4       3.3   3.7e+02    1.0   0.1     4    30   610   637   607   643 0.83 - Tetratricopeptide repeat  
G01012\_P             -           1268 TPR\_14               PF13428.1     44   0.00048   19.3   3.6   3   4       4.2   4.7e+02    0.6   0.0    19    38   771   790   769   792 0.88 - Tetratricopeptide repeat  
G01012\_P             -           1268 TPR\_14               PF13428.1     44   0.00048   19.3   3.6   4   4      0.21        23    4.7   0.0     4    28  1012  1036  1010  1051 0.89 - Tetratricopeptide repeat  
G01012\_P             -           1268 TPR\_19               PF14559.1     68    0.0067   15.4   0.2   1   3      0.35        43    3.2   0.0     4    45   410   470   408   479 0.62 - Tetratricopeptide repeat  
G01012\_P             -           1268 TPR\_19               PF14559.1     68    0.0067   15.4   0.2   2   3       1.6   1.9e+02    1.1   0.0    25    49   644   668   629   674 0.84 - Tetratricopeptide repeat  
G01012\_P             -           1268 TPR\_19               PF14559.1     68    0.0067   15.4   0.2   3   3     0.082        10    5.2   0.0    31    53  1015  1037  1008  1050 0.84 - Tetratricopeptide repeat
